# Exercise and Nutrition Strategies to Counteract Sarcopenic Obesity

**DOI:** 10.3390/nu10050605

**Published:** 2018-05-12

**Authors:** Inez Trouwborst, Amely Verreijen, Robert Memelink, Pablo Massanet, Yves Boirie, Peter Weijs, Michael Tieland

**Affiliations:** 1 Faculty of Sports and Nutrition, Amsterdam University of Applied Sciences, 1097 SM Amsterdam, The Netherlands; i.trouwborst@maastrichtuniversity.nl (I.T.); a.verreijen@hva.nl (A.V.); r.g.memelink@hva.nl (R.M.); p.j.m.weijs@hva.nl (P.W.); 2 Medical Intensive Care Unit, Nimes University Hospital, place du Pr Debré, 30029 Nimes, France; pablo.lucas.massanet@chu-nimes.fr (P.M.); yves.boirie@inra.fr (Y.B.); 3 Unité de Nutrition Humaine, Université Clermont Auvergne, INRA, CRNH Auvergne, CHU Clermont-Ferrand, Service Nutrition Clinique, F-63000 Clermont-Ferrand, France; 4 Department of Nutrition and Dietetics, Internal Medicine, VU University Medical Center, De Boelenlaan 1117, 1081 HV Amsterdam, The Netherlands

**Keywords:** sarcopenic obesity, aging, nutrition, exercise, body composition

## Abstract

As the population is aging rapidly, there is a strong increase in the number of individuals with chronic disease and physical limitations. The decrease in skeletal muscle mass and function (sarcopenia) and the increase in fat mass (obesity) are important contributors to the development of physical limitations, which aggravates the chronic diseases prognosis. The combination of the two conditions, which is referred to as sarcopenic obesity, amplifies the risk for these negative health outcomes, which demonstrates the importance of preventing or counteracting sarcopenic obesity. One of the main challenges is the preservation of the skeletal muscle mass and function, while simultaneously reducing the fat mass in this population. Exercise and nutrition are two key components in the development, as well as the prevention and treatment of sarcopenic obesity. The main aim of this narrative review is to summarize the different, both separate and combined, exercise and nutrition strategies so as to prevent and/or counteract sarcopenic obesity. This review therefore provides a current update of the various exercise and nutritional strategies to improve the contrasting body composition changes and physical functioning in sarcopenic obese individuals.

## 1. Introduction

The world population is aging rapidly. In 2050, it is predicted that about 22% of the total population will be older than 60 years, and around 5% will be older than 80 years [1]. In line with the aging of society, the prevalence of different health problems rapidly increases. In the western world, about 42% of older adults (≥55 years) have problems with performing activities of daily living and approximately 85–90% need medication for chronic diseases, such as type II diabetes, high blood pressure, or cardiovascular disease [2,3]. As a result, the risk of falls, institutionalization, loss of independence, and premature death has gradually increased in the last decennia. In addition, these burdens increase the demand on our health care system and result in a tremendous health care cost.

The contributing factors to the physical limitations with increasing age are highly multifactorial. One major contributor, however, is the reduced skeletal muscle mass with age, which is accompanied by the impaired skeletal muscle function, which is referred to as sarcopenia [4]. Obesity, or the increase in fat mass, is another major contributor to physical limitations in older adults [5]. The coexistence of sarcopenia and obesity, known as sarcopenic obesity, have an even more detrimental effect on physical limitation, as they act in a synergistic manner [6,7]. Furthermore, sarcopenic obesity has also been reported to increase the risk for metabolic disturbances, which is detrimental for several cardio-metabolic chronic diseases [8]. The prevention and/or treatment of sarcopenic obesity is therefore of major relevance for public health and individual healthy aging. Exercise strategies have been largely linked to improvements in the parameters of sarcopenia and obesity. Additionally, nutritional strategies have the potential to the improve body composition parameters in sarcopenic and obese older adults. The main aim of this narrative review is to provide a current update of the various exercise and nutritional strategies, so as to prevent and/or counteract sarcopenic obesity in older adults.

## 2. Methods Narrative Review

PubMed and Google Scholar were searched in order to identify relevant articles. The search was performed between June 2017 and March 2018. The search strategy consisted of the Boolean operator “AND”, so as to combine the following concepts:

- Sarcopenic obesity, body composition, and aging
- Nutrition and diet
- Exercise and physical activity

All of the relevant keyword variations that were used for these main concepts in the search strategy used the Boolean operator “OR”. The search results were limited to human nutrition and/or exercise intervention studies, which were aimed at improving the body composition and physical performance parameters that were related to sarcopenic obesity. Acute (one-day) studies were not included. Studies with older adults were included, which were defined as individuals above the age of 55 years old. Both English and Dutch articles were included.

## 3. Defining Sarcopenic Obesity

Sarcopenic obesity has been defined as a combination of low skeletal muscle mass and high fat mass or bodyweight, which is also illustrated in the definitions that are used for both of the conditions [9]. Different methods are used to characterize both sarcopenia and obesity. For instance, obesity has been defined using the body mass index (BMI), skinfold measurements, or fat mass [10,11,12,13,14], and sarcopenia has been defined using skeletal muscle mass [10,15,16] or skeletal muscle mass, combined with force and/or performance [4,17].

Difficulties with defining sarcopenic obesity have been mainly as a result of contrasting body composition changes. While body weight and BMI remain relatively unchanged with increasing age, the absolute skeletal muscle mass is decreased and the (visceral) fat mass is increased [4]. The sole use of weight or BMI the for diagnoses of sarcopenic obesity can therefore lead to a misinterpretation of the condition [18]. Potentially, misaligned treatment methods may be applied, which may lead to a worsening of the condition. Importantly, defining sarcopenic obesity should always include a combination of methods, including measuring body fat, skeletal muscle mass, and ideally also muscle strength. Sarcopenic obesity is an age-related disease and will therefore, in this review, be defined as present in older adults, which includes individuals at the age of 55 years and older.

The prevalence of sarcopenic obesity varies according to the definitions and the methods that are used for this definition. Using the dual-energy X-ray absorptiometry (DXA), the prevalence of sarcopenic obesity was 2% between individuals 60–69 years of age, and 10% for individuals over 80 years of age [10]. When using the Relative Skeletal Muscle Index (RSMI), 8.9% of men and 7.1% of women were sarcopenic obese [19], but others, which used the appendicular lean mass (ALM)/BMI, found an even higher variable prevalence in the U.S. population, from 16% to 40% [20].

These differences in defining the sarcopenic obesity leads to difficulties in comparing the effectiveness of the strategies that target the sarcopenic obesity. Therefore, this review focuses primarily on the improvements in skeletal muscle mass, muscle strength, physical performance, fat mass, and waist circumference, which are the parameters that are considered important when studying sarcopenic obesity [6].

## 4. Etiology of Sarcopenic Obesity

### 4.1. Age-Related Changes in Body Composition

#### 4.1.1. Skeletal Muscle Tissue

Aging is strongly related to changes in body composition. Both the loss of skeletal muscle mass and the increase in adipose tissue are common features with aging [10,21]. The gradual decline in skeletal muscle mass is accompanied with the loss of muscle strength and physical performance, which is also described as sarcopenia [22]. The development of sarcopenia is partially as a result of low levels of physical activity and inadequate nutritional intake [4]. Furthermore, several age-related changes, such as endocrine disturbances, mitochondrial dysfunction, and neuro-degenerative diseases, can contribute to the development of sarcopenia with age [4].

Already at the age of 30, the skeletal muscle mass starts to decline, with more significant losses after the age of 65 and 80 years [23]. A quantitative review has shown that the median decline in skeletal muscle mass throughout the lifespan is 0.37% and 0.47% per year in women and men, respectively, with even higher rates in people aged 75 or over [24]. Different studies have reported a substantial decrease in the muscle fiber size in the older adults, which accompanies this loss in the skeletal muscle mass [25,26,27]. Emerging evidence, however, shows that the decline in skeletal muscle mass is not the sole contributor to the decline in physical performance. A variety of factors, such as disturbances in motor coordination, excitation-contraction coupling, energetics, skeletal integrity, fat infiltration, and decreased skeletal muscle aerobic capacity, are important for physical performance in the older adults [28,29].

#### 4.1.2. Adipose Tissue

A peak in the adipose tissue mass is generally observed around the age of 65 and is mainly characterized by an increase in the visceral fat mass [30,31], which is highly associated with the development of obesity [32]. Increased visceral fat is an important risk factor for many health conditions, such as type II diabetes, ischemic heart disease, hypertension, and certain cancers, all of which contribute to a decreased quality of life and premature mortality [33].

It was estimated that the prevalence of obesity in Europe in adults, aged 60 years or older, ranged from 20 to 30 percent in 2015 [34]. Obesity is caused by an imbalance between the energy intake and energy expenditure. High caloric intake and low levels of physical activity could, therefore, largely contribute to the development of obesity [35]. However, the reduction in the oxidative capacity as a result of the loss of skeletal muscle or the reduced mitochondrial function, may also contribute to the fat accumulation in the body and also within the skeletal muscle, that is, ectopic lipid deposition [36]. In addition, hormonal changes, such as a decrease in growth hormone and testosterone secretion, reduced thyroid hormone responsiveness, and leptin resistance, are commonly seen with age and could, via different mechanisms, contribute to the development of obesity [37]

### 4.2. Causes and Consequences of Sarcopenic Obesity

Since older adults are at risk for both the development of sarcopenia and obesity, a double burden exists. This is a condition that is defined as sarcopenic obesity [38]. Since, by definition, sarcopenic obesity is a combination of two conditions, the consequences of sarcopenic obesity largely overlap with both sarcopenic and obesity. A few, but not all, of the consequences include an increased risk for physical limitations, hypertension, cardiovascular disease, type 2 diabetes, and dyslipidemia [39].

Interestingly, older adults with low skeletal muscle mass and strength are 1.95 to 2.62 times at risk of being obese, compared with older adults with normal skeletal muscle mass [40]. This could be explained by the risk factors for the development of sarcopenia and obesity, as they are often similar [8,40]. This makes it not very surprising that the two conditions often coexist. Additionally, it is often thought that sarcopenia and obesity work in a synergistic manner, as the consequences of sarcopenic obesity are often more severe than for sarcopenia or obesity alone [6,7].

An important risk factor for both sarcopenia and obesity is the lower rate of energy expenditure with age, which is a result of lower physical activity, as well as a fat free mass related-lower basal metabolic rate, which is often seen with older age [35,41]. Furthermore, the physiological factors that are associated with age, such as changes in hormone levels, vascular changes, low grade inflammation, and immunological factors, could contribute to the development of both sarcopenia and obesity [42]. Therefore, it is not very surprising that the two conditions often coexist.

Not only do sarcopenia and obesity have similar pathological causes, but the physiological consequences of obesity are also risk factors for the development of sarcopenia [43]. To illustrate this, obesity may cause the resistance to anabolic stimuli, such as, growth factors, hormones, amino acids, and exercise, a phenomenon called ‘anabolic resistance’ [44]. The increase in intramuscular fat is an important factor leading to anabolic resistance, by affecting the signaling pathways that are involved in the muscle protein synthesis, and thereby increasing the risk for sarcopenia [45,46,47]. Furthermore, free fatty acids [48] and insulin resistance are metabolic reasons for the anabolic resistance in the muscle of older subjects [49].

**Figure 1:**
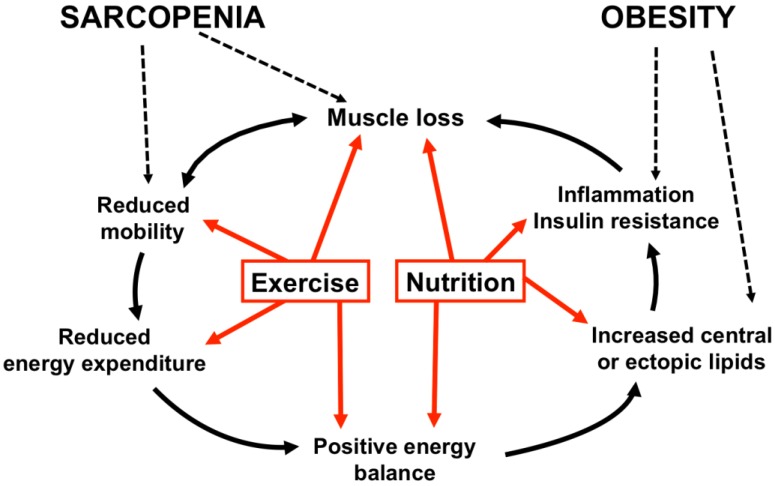
Pathophysiology and interventions in sarcopenic obesity. Black arrows indicate the pathophysiology of sarcopenic obesity. Red arrows indicate potential targets of nutritional and exercise interventions to counteract sarcopenic obesity.

In addition, obesity is responsible for causing systemic low-grade inflammation, particularly by visceral fat, which excretes several different pro-inflammatory cytokines, such as interleukin-6 (IL-6) and TNF-α [50,51]. Obesity-induced low-grade inflammation also has the potential to contribute to anabolic resistance, leading to several cardiovascular and metabolic complications, such as insulin resistance [52,53]. Finally, inflammation and insulin resistance have differential influences on muscle metabolism, both inhibiting the protein synthesis. However, the activation of proteolysis is mostly stimulated by inflammation [54]. Finally, the obesity-induced muscular fat infiltration does not only accelerate the anabolic resistance in obese older adults, but it also affects the muscle quality. The storage of adipose tissue in the muscle leads to an increased stiffness of the muscle, affecting the shortening and expansion capacity of the muscle fiber [55]. This leads to a decrease in the force per unit of the skeletal muscle tissue, and thus a decrease in the muscle quality [56]. In summary, there are multiple causes and consequences for sarcopenic obesity, which are interrelated (Figure 1). Although some causes and consequences are not completely elucidated, effective nutritional and exercise strategies to counteract sarcopenic obesity are certainly needed.

## 5. Exercise Strategies

Exercise has been found to be an effective strategy for treating different conditions in various populations, such as cardiovascular disease, pulmonary disease, diabetes, and several cancers [57]. In addition, exercise as a strategy to prevent or treat obesity, has often been proposed in the literature and it has been widely studied in human intervention studies [58]. In addition, exercise is a widely used strategy in order to improve muscle mass, muscle strength, and physical performance in (sarcopenic) older adults [59].

The potential mechanisms by which exercise can induce improvement in the sarcopenia and obesity parameters are multi-factorial. Firstly, exercise has an important role in regulating energy balance. The energy costs that are accompanied with exercise could, in combination with a hypocaloric diet, contribute to a lower energy balance. Consequently, exercise is often a component of strategies that target the loss of fat mass in obese older adults [60,61,62,63,64]. Furthermore, exercise can improve the physical functioning parameters, such as hand-grip strength, gait speed, balance, and aerobic capacity, in both sarcopenic and obese populations [65]. Finally, exercise is, together with nutritional intake, the main anabolic stimulus which leads to a muscle protein synthetic response [66]. Although the muscle protein breakdown is also stimulated with exercise, in the fed state, the net balance of the protein breakdown and synthesis is increased after exercise, which leads to muscle protein gain and thus muscle hypertrophy [67,68]. It should be noted that the main goal is to improve mobility and autonomy of the obese sarcopenic patients, by improving elasticity, strength, and muscular endurance. Exercise prescription should take into account the intensity, volume, frequency, and progression of training. Since exercise is an effective strategy to improve the body composition parameters in both sarcopenia and obesity, exercise, as a strategy to counteract sarcopenic obesity, is extensively discussed in the literature. Below, the effect of the different types of exercise on the sarcopenic obesity parameters will be discussed, namely, resistance, eccentric, aerobic, concurrent, and electro exercise. Table 1 provides an overview of the different studies that have investigated the effect of various exercise modalities in sarcopenic obese individuals.

### 5.1. Resistance Exercise

Resistance exercise is currently seen as the most effective exercise strategy in order to elicit muscle hypertrophy and to improve muscle function and strength in older adults [69,70,71,72]. The majority of the studies have been performed among older, non sarcopenic obese adults. For example, a meta-analysis of 49 studies, with in total 1328 participants of 50 years and over, showed that, on average, the skeletal muscle mass was increased by 1.1 kg (95%CI: 0.9–1.2, *p* <0.01) after an average of 20.5 weeks of resistance training two to three times a week [71]. Another meta-analysis of Peterson and colleagues also illustrated the effect of resistance exercise on muscle strength in a total of 1079 older adults, where an average of 17.6 weeks led to muscle strength improvements of up to 33% (SE: 2%, *p* < 0.01), depending on the type of muscle [70]. As such, resistance exercise is a powerful strategy to counteract sarcopenia in older adults.

In a sarcopenic obese population, the effect of resistance exercise on body composition and skeletal muscle function is less established. Vasconcelos et al. [73] showed that a 10 week resistance exercise program was not effective to improve physical function (−0.14, 95%CI: −1.04–0.76), strength (−6 J/kg, 95%CI: −0.90–12), or power (−13 w/kg, 95%CI: −1.4–28) in older women with sarcopenic obesity, compared with the non-exercising control group. The relatively short duration of the intervention and the small sample size may have contributed to this result. Gadelha et al. [74], however, demonstrated improvements in both strength (12.42 N, SE: 1.18, *p* < 0.001) and skeletal muscle mass (0.29 kg, SE: 0.11, *p* < 0.001), compared to the control group, after 24 weeks of traditional resistance training. Furthermore, a recent 12 week intervention-study examined the effect of elastic band resistance exercise in sarcopenic obese older women, and showed that skeletal muscle mass (0.73 kg, 95%CI: 0.08–1.39, *p* < 0.05), muscle quality (2.63 kg/kg, 95%CI: 1.21–4.05, *p* < 0.01), and physical capacity (8.58, 95%CI: 4.79–12.36, *p* < 0.001) were significantly improved, compared to the no exercise group [75]. Similar results were found in another program that wasinvolved in an elastic band training for 12 weeks in sarcopenic obese older women, which, in addition, also demonstrated a significant decrease in fat mass (−0.58, *p* = 0.035), compared with the no exercise group [76]. Furthermore, another recent study reported that 8 weeks of resistance exercise in 60 sarcopenic obese older adults resulted in the maintenance of skeletal muscle mass (0.1 kg, *p* < 0.05), decreased fat mass (−1.0 kg, *p* < 0.05), and increased grip strength (3.5 N/kg, *p* < 0.05), compared with the non-exercise group [77]. Overall, the majority of the mentioned studies showed that resistance exercise is an effective strategy to improve the body composition in sarcopenic obesity, and that it has the potential to improve physical performance.

### 5.2. Eccentric Exercise

Eccentric exercise is a different approach of the concentric contraction of the skeletal muscle, where the muscle contracts while stretching itself (for instance during stairs descent). This type of muscular work has the advantage of increasing the muscle strength [78] with a reduced energy consumption in comparison with the concentric contraction [79]. This strategy has already been used in the rehabilitation of various groups of patients, such as diabetics, neurological diseases, and groups of patients with cardiorespiratory pathologies [80]. A study that was done in a geriatric population (14 women and 14 men, mean age 80 years, without sarcopenic obesity), compared a resistance exercise and a surplus exercise strategy. The patients were followed for 12 weeks, with body composition assessment (DEXA) and muscle biopsies done before and after the 12 weeks of training. The eccentric exercise improved the strength (1.3 N/kg *p* < 0.05) and body composition (fat reduction of −3.1% vs. −0.2% [*p* < 0.05]), in comparison to concentric strategy [81]. Another study compared two strategies of exercise, namely, a strategy with a combination of aerobic and eccentric exercise (A/E) with a purely aerobic strategy (A), for 16 weeks in a population of type 2 diabetic patients. The BMI average of the A/E group was 35 kg/m^2^, but individuals did not per se have sarcopenia. Both of the strategies improved the glycaemic control, intramuscular fat, and physical performance (6 min walk test). The A/E group had a more marked decrease in BMI (−1.9 kg/m^2^, 95%CI: −3.2—0.6, *p* < 0.05), with an increase in the lean body mass (15.1 cm^2^, 95%CI: 7.6–22.5, *p* < 0.05) [82]. The listed studies did not include the sarcopenic obese individuals, nonetheless, the eccentric exercise could potentially be an effective strategy for this population, as it simultaneously targets the fat mass and skeletal muscle mass.

### 5.3. Aerobic Exercise

Aerobic exercise may be an important strategy to improve the muscle function, by enhancing the muscle aerobic capacity in older adults [83,84]. Aerobic exercise has the potential to improve aerobic capacity by initiating mitochondrial adaptation [85], enhancing cardiovascular function (e.g., increased stroke volume capacity) [86] and increasing the capillary density of the muscle tissue [87]. Moreover, aerobic exercise training could have beneficial effects on body fat mass, especially in combination with a dietary intervention, and it is therefore also seen as an effective strategy to counteract the development of obesity [88,89].

The separate effects of aerobic exercise on the muscle aerobic capacity in sarcopenic older adults and its effect on fat mass in obese older adults (although small [90]), are largely supported by the literature. This leads to the expectation that aerobic exercise could also improve these parameters in the sarcopenic obese patients. However, to our knowledge, only one study has investigated the effect of aerobic exercise specifically in a sarcopenic obese population. This 8 week randomized controlled trial included 60 sarcopenic obese older adults (aged 65–75), and found that aerobic exercise significantly led to improvements in body fat mass (−0.7 kg *p* < 0.05) and visceral fat (−6 cm^2^, *p* < 0.05), and maintained the skeletal muscle mass (+0.1 kg, *p* < 0.05), compared to the control group [77], making it a promising strategy.

Although there is only limited evidence available on the effects on aerobic exercise on sarcopenic obesity, aerobic exercise seems like an effective tool for losing excess fat mass and improving muscle performance in sarcopenic obese, older adults. Nonetheless, aerobic exercise, in combination with other strategies, such as resistance exercise or a nutritional strategy, could potentially be more effective in targeting sarcopenic obesity.

### 5.4. Concurrent Exercise

Concurrent exercise is the combination of both resistance exercise and aerobic exercise. Both resistance exercise and aerobic exercise have potentially positive effects on several body composition parameters in sarcopenic obesity, and could improve the muscle function. Furthermore, in addition to this effect, the combination of these strategies, called concurrent exercise, can also promote the loss of fat mass. Concurrent exercise may therefore be an important strategy to improve the skeletal muscle mass and function, and simultaneously support the loss of fat mass in sarcopenic obese older adults.

A randomized controlled trial with 45 healthy adults found that concurrent training six days a week for 12 weeks, resulted in similar improvements in maximal oxygen consumption (VO_2max_), compared to aerobic exercise alone, but the improvement in the knee extension one repetition maximum (1 RM) was lower than in the resistance exercise group alone (10.0 kg vs. 8.2 kg, *p* < 0.05) [95], and similar results were found in elderly men (*n* = 60) [96]. These results indicate that aerobic exercise potentially leads to a blunted hypertrophic response to resistance exercise in non-sarcopenic obese individuals. Villareal et al. [64], however, showed that the combination of both aerobic and resistance exercise during a weight management program was more effective in improving the functional status of obese older adults, than resistance or aerobic exercise alone. In this clinical trial with 160 obese older adults, the physical performance test (PPT) score improved more in the concurrent exercise group than in the separate exercise groups (21% increase versus 14% in both the aerobic and resistance group [*p* < 0.05]). Additionally, the muscle mass was best preserved after combined exercise (−3%) and resistance exercise (−2%), compared with aerobic exercise (−5%) (*p* <0.05). There was one study available that was studying the effect of concurrent exercise in saropenic obese individuals, and it reported findings in line with Villareal et al. [64]. In this randomized controlled trial with 139 sarcopenic obese women, it was reported that 3 months of bi-weekly, 60-min concurrent exercise, had resulted in a 17.8% (SE: 4.2, *p* = 0.119) increase in knee extension strength (N), a significant increase in arm (1.8%, SE: 0.6, *p* < 0.05) and leg muscle mass (2.2, SE: 0.7, *p* < 0.05), and a decrease in the total body fat mass (−5.5%, SE: 0.9, *p* < 0.05), compared with the control group (no exercise) [91]. Although limited evidence is available, these results illustrate the potential beneficial effect of concurrent exercise on sarcopenic obesity.

### 5.5. Electro Stimulation

Electrostimulation has recently become a popular technic in order to simulate skeletal muscular contraction. This method may be an alternative way for obese and sarcopenic patients to perform physical activity. A study in women older than 70 years compared electrostimulation, with and without protein treatment, and a control group [97]. This feasibility study showed that electrostimulation could be applied in this population, because it had a positive effect on the waist circumference, and it improved blood pressure, the latter being important for improving the metabolic syndrome. Specifically in a sarcopenic obese population, electrostimulation was compared with a control group without intervention. It was demonstrated that the electrostimulation had a stronger decrease in the total body fat (−2.05%, 95%CI: −1.40—2.68, *p* < 0.001) and an increase in the muscular strength (as measured by handgrip) (1.90 kg, 95%CI: 0.99–2.82, *p* < 0.001), compared to the control group [92]. No adverse effect in patients that were treated with electrostimulation was detected, meaning that it was well tolerated. So the question of whether electrostimulation is a suitable method for working with patients who are not able to do physical activity, is still being debated.

## 6. Nutritional Strategies

Nutrition is a key factor in the development of both sarcopenia and obesity. The mechanisms by which nutrition affects sarcopenia and obesity are, however, different. Sarcopenia is associated with an inadequate nutritional intake, whereas obesity is a result of an excess consumption of energy, leading to an imbalance between the energy intake and energy expenditure [40]. Designing nutritional strategies for sarcopenic obesity should target both an optimal nutrient intake, so as to increase skeletal muscle mass or prevent muscle mass loss, as well as an optimal nutrient and energy intake to decrease excess fat mass. The key question is how we can preserve muscle anabolism in a situation of energy deficit, in order to avoid a high proportion of weight loss as fat free mass in this population that is prone to muscle loss.

### 6.1. Hypocaloric Diets

A hypocaloric diet is an energy restriction diet that aims at losing body weight. The most optimal and safe range of energy restriction for sarcopenic obese older adults is an energy deficit of about 200–700 kcal per day [98]. There is ample data available that indicates that hypocaloric diets are very effective for losing weight in obese older adults [99,100,101]. However, although an energy restricted diet in obese older adults leads to the loss of fat mass, this is often accompanied by the loss of skeletal muscle mass [102,103,104]. For instance, a study of Villareal et al. [63] reported that an average fat mass loss of 7.1 kg (SE: 3.9, *p* < 0.001) in obese older adults following a hypocaloric diet (−500 to −750 kcal/day) for 52 weeks. However, this fat mass loss was accompanied with an additional 3.2 kg (SE: 2.0, *p* < 0.001) skeletal muscle mass. It is estimated that about 25 percent of the weight loss that is achieved with hypocaloric diets in obese older adults is skeletal muscle mass [34,105,106,107]. Especially for sarcopenic obese older adults, the loss of skeletal muscle mass is highly detrimental for retaining the ability to walk or climb stairs. Apart from the accompanied skeletal muscle mass loss with hypocaloric diets, solely focusing on losing weight can also have harmful effects for the micronutrient status [108] and bone mineral density [109], and is therefore highly undesirable. A weight loss diet in this population should therefore always focus on the preservation of muscle mass and could be combined with a high protein diet and/or micronutrient supplementation.

### 6.2. Protein Intake

It is well established that the intake of dietary amino acids, and especially the essential amino acids (EAA), has a positive regulatory effect on the muscle protein synthesis in the muscle [110,111,112]. However, the protein synthetic response to the anabolic stimuli, such as dietary protein intake, is blunted in older adults [113,114] and in obese individuals [44]. As a result, obese older adults may have higher protein needs compared with younger lean people, in order to optimally promote muscle protein synthesis so as to maintain or to regain muscle proteins [115]. As a result, the recommended intake of 0.8 g/kg body weight (BW) for healthy adults may not be sufficient to meet the protein needs in older (obese) adults [116]. To maintain and regain the muscle mass and function in the long term in older people (>65 years), it is recommended to have a dietary protein intake of 1.0 to 1.2 g/kg BW with even a higher intake (1.2–1.5 g/kg BW), especially for individuals that suffer from chronic diseases [117]. A dietary intervention study with 104 sarcopenic obese older adults (>65) showed that a 3-month hypocaloric diet high in proteins (1.2 g/kg BW) led to a small increase in the muscle mass index (0.2, SE: unknown, *p* < 0.01), whereas a hypocaloric diet that was low in protein (0.8 g/kg) led to a significant decrease in the muscle mass index (−0.2, SE: unknown, *p* < 0.01) [93]. Although the long-term intervention studies that are investigating the optimal protein intake for the sarcopenic obese individuals are lacking, a minimal intake of 1.0–1.2 g/kg BW seems essential in order to maintain muscle mass in this population, so as to compensate for the anabolic resistance as is present in sarcopenic obesity, especially in periods of energy deficit.

The type of protein and the amino acid composition are also suggested as being relevant for muscle mass preservation or gain during weight loss. Whey protein, a milk derived protein, has been shown to be very effective in stimulating postprandial muscle protein accretion in older men [118,119], which has been ascribed to its fast digestion and absorption kinetics, and the high leucine content [115,120]. In addition, an intake of about 2.0–2.5 g/day leucine, which has been mainly derived from animal sources, improves the post-prandial muscle protein synthesis in elderly men [121]. Furthermore, although it has been suggested by some, the differential effect of the different protein sources (plant or animal) on fat mass loss or gain, has not been fully confirmed [122]. Overall, dietary protein that is derived from animal source products, rather than from plant-based sources, seems most effective in eliciting muscle protein synthesis [123]. Although it has not yet confirmed in sarcopenic obese individuals, higher intakes of animal source protein might contribute to improved muscle mass in this population.

Furthermore, the timing of protein intake seems important in order to optimally promote muscle protein synthesis. A study in which the dietary intake of in total 1279 elderly was analysed, showed that about 80 percent of the dietary protein was consumed during the three main meals, of which the most was during dinner [124], also referred to as the ‘pulse diet’ [125]. Interestingly, a more evenly distribution of dietary protein intake, that is, every 3 to 4 h (the ‘spread diet’), led to a higher protein synthesis rates (25%, *p* = 0.003) [126], and is associated with higher muscle strength, physical performance and skeletal muscle mass in older adults [127,128,129]. Additionally, although only preliminary data was present, increasing the number of meals per day, may have stimulated the overall satiety, prevented excess food intake, and therefore potentially reduced the obesity risk [130]. Thus, merely focusing on the total amount of dietary protein may not be most optimal for improving the parameters of sarcopenic obesity, as a more spread protein intake during the day may also be an important factor that augment the effect of its intake.

Another strategy would be to combine several anabolic nutrients, such as protein, amino acids, vitamin D, and omega 3. Indeed, recent studies have shown that combining whey protein, which has been enriched with leucine and with vitamin D, could increase protein synthesis and finally promote muscle mass gain in older adults [131,132]. It was reported that this combination was also effective in adults (35–65 years) [118] and in obese older adults [133].

In summary, the sufficient intake of dietary proteins is essential in sarcopenic obese older adults because of the blunted anabolic responses. The anabolic response to the dietary intake may be amplified by the relatively high intakes of animal source protein, which contain the amino acid leucine, in each meal and may be amplified by a more evenly distributed dietary protein intake over the day (spread diet). Strategies that aim to increase the skeletal muscle mass and function, by optimizing muscle protein synthesis in sarcopenic obesity, should therefore take these factors into account.

### 6.3. Micronutrients

The low intake and low status of several micronutrients have been linked to the development of sarcopenia in older adults. Although mainly observational studies are available, minerals, and particularly, magnesium, selenium, and calcium, seem to be most promising to counteract or prevent sarcopenia [134]. Furthermore, low 25-hydroxy-vitamin D status is associated with the development of sarcopenia [135]. In addition, a systematic review of Muir and Montero-Odasso [136] showed that the supplementation of vitamin D, with daily doses of 800 to 1000 IU, improves several sarcopenic parameters in older adults. Overall, the micronutrient deficiencies predict the development of frailty and sarcopenia in older adults [137]. Individuals that are at risk for micronutrient deficiencies, should therefore focus on improving the micronutrient status, in order to potentially prevent the development of sarcopenia.

Especially in obese adults, the risk of developing micronutrient deficiencies is relatively high. Low concentrations of vitamin B6, vitamin C, 25-hydroxyvitamin D, vitamin E, selenium, magnesium, and zinc are found in several obese populations, compared with normal-weight adults [138,139,140]. In addition, obese individuals following a weight loss diet are especially at risk for micronutrient deficiencies [108]. Although causal evidence is lacking, the obesity related deficiencies in several micronutrients are also linked to a decline in muscle mass, strength, and physical performance [141], and thereby potentially worsening the sarcopenic outcomes. To achieve an adequate micronutrient intake during a low caloric intake, nutrient dense food products or micronutrients supplements may be warranted.

### 6.4. Hypocaloric Diet with High Protein Intake

Each of the strategies that are discussed above are individually not effective in targeting all of the sarcopenic obesity parameters simultaneously. A hypocaloric diet induces fat mass loss, but this is accompanied with the loss of the skeletal muscle mass, and protein intake, which promotes the muscle protein synthesis, but is not effective in addressing the obesity parameters. An optimal strategy should therefore combine different strategies in order to optimize its effect and target both fat mass, as well as skeletal muscle mass, muscle strength, and physical performance. Below, the combination of a hypocaloric diet with a high protein intake will be discussed as a potentially effective strategy.

In a meta-analysis, Kim, et al. [142] analyzed the effects of protein intake (<25% vs. ≥25% of energy intake or ≥1.0 g/kg/day) during a hypocaloric diet on the changes in body mass, skeletal muscle mass, and fat mass in older adults. They demonstrated that the older adults preserved more skeletal muscle mass (0.83 kg, 95%CI: 0.47–1.19, P: unknown) and lost more fat mass (−0.53 kg, 95%CI: −1.08 to 0.03, P: unknown) during weight loss when consuming higher protein diets (intake ≥1.0 g/kg/day), compared with a lower protein diet. Although this seems a promising result for the treatment of sarcopenic obesity, inconsistent results were found in the obese and physically limited older adults, where the effect of a high protein hypocaloric diet using meal-based protein foods over a 6 month period was evaluated [143]. The intervention group received individual hypocaloric dietary advice (500 kcal below needs) incorporating ≥30 g protein in each meal (mainly animal protein), which resulted in a protein intake of 1.2 g/kg/day. They found a positive effect on physical performance, but no significant effect on skeletal muscle mass. Furthermore, Backx, et al. [144] randomized the overweight and obese older adults into either a high protein diet group (1.7 g/kg/day) or into a normal protein diet group (0.9 g/kg/day) during a 12 week hypocaloric diet. No preservation of muscle mass (−1.8 kg, SE: 2.2) or muscle strength (−8.8 kg, SE: 14.0) was observed in those who were consuming a high protein diet during a caloric restriction (with no differences between diets for muscle mass: *p* = 0.213 and muscle strength: *p* = 0.689). Thus, although the combination of a hypocaloric high-protein diet seems to be effective in the prevention of sarcopenic obesity, this strategy does not seem to be effective for the treatment of sarcopenic obesity.

## 7. Combined Nutrition and Exercise Strategies

A one component strategy, whether it is an exercise or a nutritional strategy, may not be the most effective in countering sarcopenic obesity. The combination of strategies, in order to target all of the aspects of sarcopenic obesity, seems to be the most appropriate. Different studies that have used a combination of strategies so as to prevent or treat sarcopenic obesity will be discussed below.

### 7.1. Hypocaloric Diet and Exercise

A hypocaloric diet in obese older adults is often accompanied with the loss of skeletal muscle mass. Strategies that preserve muscle mass while simultaneously stimulating the loss of fat mass, are therefore essential in the treatment of sarcopenic obesity. Adding exercise to a hypocaloric diet in sarcopenic obese older adults could potentially prevent the loss of skeletal muscle mass, by its muscle protein stimulating response. Furthermore, not only muscle mass, but also muscle quality (strength and performance), can be improved following exercise. A hypocaloric diet in combination with exercise, in order to counteract sarcopenic obesity, has therefore been largely discussed in the literature [63,107,145,146].

To illustrate this, the effect of progressive resistance exercise that was added to an energy restriction diet for 6 months, was studied in 30 frail obese older adults (70 ± 5 years), compared with only an energy restriction diet [94]. They found that the total fat mass was reduced in both of the groups, but the loss of skeletal muscle mass was reduced in the diet and exercise group, compared to the diet group (−1.8, SE: 1.5 vs. −3.5, SE: 2.1 kg, *p* < 0.05). In addition, the diet and exercise group had up to a 43% increase in strength (SE: 45, *p* < 0.05), whereas the diet group had maintained strength. Similar results were found in a study of Villareal et al. [63], with 107 frail obese older adults. The authors evaluated the effect of a 1 year multi-component exercise program with and without a hypocaloric diet (−500 to 750 kcal per day). The multi-component exercise program, consisting of aerobic, resistance, balance, and flexibility exercises, in combination with the hypocaloric diet, led to greater improvements in the physical function that was assessed by the PPT score (5.4, SE: 2.4, *p* = 0.04) and it led to a significantly improved (*p* < 0.001) preservation of the skeletal muscle mass (−1.8, SE: 1.7 kg), compared with the diet group (−3.2, SE: 2.0 kg). This finding is in line with a systematic review, which reported that the addition of exercise to energy restriction in obese older adults attenuated loss of skeletal muscle mass on average from 24% to 11% [107].

A very recent and key study of Villareal et al. [64] evaluated the separate and combined effects of resistance and aerobic training, in combination with a hypocaloric diet, in 160 frail obese older adults. After 6 months, the body weight had decreased by 9% (SE: 0.9 kg, *p* < 0.001) in all of the exercise groups, and did not change significantly in the control group (no weight-management or exercise program). The skeletal muscle mass (SMM) decreased less and the strength increased more in the combination and resistance groups (SMM: −3% [*p* = 0.047] and −2% [*p* < 0.001], respectively, strength: +18% [*p* < 0.001] and +19% [*p* < 0.001], respectively) than in the aerobic group (SMM: −5%, strength: +4%), whereas the peak oxygen consumption increased more in the combination and aerobic groups (+17%, *p* < 0.001 and +18%, *p* < 0.001, respectively) than in the resistance group (+8%). The physical performance test score increased more in the combination group (+21%) than in the aerobic (+14%, *p* = 0.002) and resistance groups (+14%, *p* = 0.004). The authors concluded that the combined aerobic and resistance exercise was the most effective for improving the functional status of the obese older adults, in combination with a hypocaloric diet. However, although exercise in combination with a hypocaloric diet was more effective that either of the interventions alone, the skeletal muscle mass was not completely preserved. Especially in thesarcopenic obese older adults, it was highly important that the skeletal muscle mass was preserved and, ideally, increased.

### 7.2. Protein Intake and Exercise

It is widely studied that the combination of high protein intake or protein supplementation with resistance exercise is an effective strategy to improve muscle mass, muscle strength, and physical performance in sarcopenic older adults [147,148,149]. To our knowledge, there is only one study that has investigated the role of exercise and protein, without including a hypocaloric diet, in body composition parameters in the sarcopenic obese population [91]. This randomized controlled trial included a total of 139 sarcopenic obese older adults, who had either followed a 60 min concurrent exercise with bi-daily supplementation of 3 g essential amino acids, of which approximately 1.3 g was leucine, or had only followed the exercise intervention. The improvements were observed for the exercise with EAA supplementation in fat mass (with EAA: −5.5 kg SE: 0.9, *p* = 0.36), compared to the control group (health education). No significant improvement in the skeletal muscle mass and physical functioning were observed for both the exercise with and without the EAA. The relatively low amounts of amino acid supplementation could be explained by the absence of effects. However, based on the individual effects of protein intake and exercise on sarcopenic obesity parameters, it was expected that changes in the body composition would be found. Future studies should investigate the effect of a high protein diet combined with exercise in order to counteract sarcopenic obesity.

### 7.3. Hypocaloric Diet, Protein Intake and Exercise

In a randomized controlled trial, the effect of a high whey protein-, leucine-, and vitamin D-enriched supplement on muscle mass preservation was studied during a 13 week weight loss program, including 3 times/week resistance exercise in obese older adults [150]. Subjects in the intervention group received a supplement containing 21 g whey protein (10 servings/wk), whereas the control group received an isocaloric control supplement. In this study, the high whey protein-, leucine-, and vitamin D-enriched supplement resulted in a muscle preserving effect of 0.95 kg (+0.4, SE: 1.2 and −0.5, SE: 2.1, respectively, *p* = 0.03), compared with the control. In another study that compared a high whey protein diet (1.2 g/kg/day) with a control diet (0.9 g/kg/day maltodextrin), during caloric restriction and flexibility/aerobic exercise training, it was found that increasing the protein intake during weight loss could counteract the deleterious effects on the muscle mass by maintaining more muscle, relative to the weight that was lost. Other interesting results on the combination of high protein intake and exercise were found in a randomized controlled trial, including 100 overweight and obese adults who followed a hypocaloric diet, either combined with high protein, resistance exercise, or both. The high protein diet (1.13 g/kg/day) and resistance exercise group alone did not significantly affect changes in the fat free mass, whereas the combination of a high protein intake with resistance exercise resulted in a significant increase in the fat free mass of 0.6 kg (SE: 1.3, *p* = 0.011) [151]. Verreijen et al. [150], Mojtahedi, et al. [152], and Verreijen et al. [151] all included a training component in their studies, which might have explained the muscle preserving effect of the protein in these studies in comparison with the studies of Porter Starr et al. [143] and Backx et al. [144], as described in paragraph 5.4. However, it should be noted that these studies were not performed in a sarcopenic obese population. In summary, weight loss trials in obese older adults are effective in reducing body weight and fat mass, but they may also reduce skeletal muscle mass. Higher protein intakes could help to prevent muscle mass loss, especially when combined with an exercise intervention. Studies including sarcopenic obese individuals are highly warranted.

## 8. Final Remarks and Conclusions

To date, many different effective strategies have been developed to counteract either sarcopenia or obesity. However, only a limited number of studies have focused on the combination of both of these conditions. The aim to simultaneously increase the skeletal muscle mass and decrease fat mass may be challenging for developing effective strategies. As sarcopenic obesity has a synergistic detrimental effect on the physical functioning and overall health, effective strategies that counteract sarcopenic obesity are highly warranted. The present review aimed to summarize these effective strategies, however, combining the results in a meta-analysis was not possible as the outcome measures and designs among the studies differ tremendously, which may be seen as a limitation of this review. Additionally, because different methods are used to define sarcopenic obesity, it is difficult to compare the effectiveness of studies. Nevertheless, this review shows that sarcopenic obesity is a highly multi-factorial condition, which requires a multi-targeted approach. This review provides the latest overview of both exercise and nutrition interventions targeting both the body composition and physical functioning in sarcopenic obese individuals.

In line with this aim, this review shows that a combination of a moderate weight loss diet, with concurrent exercise and a high protein intake (≥1.2 g/kg/day), which is relatively high in animal protein and spread throughout the day, has the highest potential in improving different parameters of sarcopenic obesity. However, further research is needed to better understand the optimal rate of weight loss, the type, intensity, and frequency of the exercise, the combined effects of the different individual strategies (exercise and nutritional) on body composition and physical functioning parameters in sarcopenic obese older adults. Finally, as new interventional technics and bariatric surgery are spreading out for the treatment of obesity in adults, we may have to consider the long term impact of bariatric surgery on muscle preservation after surgery, and the optimal strategy to maintain mobility in these aging patients in the future [153].

## Figures and Tables

**Table 1:** Exercise and nutrition strategies to improve body composition and physical performance in sarcopenic obesity.

	*N* *	Age (Mean)	Sarcopenic Obesity Definition	Type of Intervention	Intervention Effect **
**Exercise strategies**					
Vasconcelos et al. [73]	28	72	BMI and HGS	10 weeks RE 2/week or no exercise	No sig. difference in SPPB (points) or muscle strength (kg)
Gadelha et al. [74]	133	67	BMI, FFM and PT	24 weeks RE 3/week or no exercise	SMM (kg): +0.29, SE: 0.11, *p* < 0.001 Strength (kg): +12.42, SE: 1.18, *p* < 0.001
Liao et al. [75]	46	67	BF% and SMI	12 weeks elastic RE or no exercise	SMM (kg): +0.73, 95%CI: 0.08–1.39, *p* < 0.05 Muscle strength (kg): +2.63, 95%CI: 1.21–4.05, *p* < 0.01 Physical capacity: +8.58, 95%CI: 4.79–12.36, *p* < 0.001
Huang et al. [76]	35	>60	BF% and SMI	12 weeks elastic RE 3/week or no exercise	FM (kg): −0.58, *p* = 0.035 SMI (FMM/m^2^): no sig. difference
Chen et al. [77]	60	69	BMI, VFA and SMI	8 weeks RE, AE, RE + AE or no exercise	FM (kg): RE: −1., AE: −0.7, RE + EA: −1.1, all with *p* < 0.05 SMM (kg): RE: +0.1, AE: +0.1, RE + EA: +0.2, all with *p* < 0.05 HGS (kg): RE: +3.5, *p* < 0.05, AE and RE + EA: no sig difference
Kim et al. [91]	139	81	BF%, SMI, gait speed and HGS	3 months CE or no exercise	Arm muscle mass (kg): +1.8, SE: 0.6, *p* < 0.05 Leg muscle mass (kg): +2.2, SE: 0.7, *p* < 0.05 FM (kg): −5.5%, SE: 0.9, *p* < 0.05 Strength (kg): +17.8%, SE: 4.2, *p* = 0.119
Kemmler et al. [92]	100	77	BMI, SMI and HGS	16 weeks electrostimulation or no exercise	FM (kg): −2.05%, 95%CI: −1.40—2.68, *p* < 0.001 HGS (kg): +1.90, 95%CI: 0.99–2.82, *p* < 0.001
**Nutrition strategy**					
Muscariello et al. [93]	104	67	BMI and SMI	3-mo hypocaloric diet with high (1.2 g/kg/bw) or low (0.8 g/kg/bw)(control diet) protein	FM (kg): no sig. difference SMI (FMM/m^2^): 0.2, SE: NP, *p* < 0.01
**Combined exercise and nutrition strategies**					
Frimel et al. [94]	30	69	BMI and PPT	6-months hypocaloric diet with or without (control) RE	FM (kg): no sig. difference FFM (kg): 1.8, SE: 1.5, *p* < 0.05 Strength (kg): up to 43%, SE: 45, *p* < 0.05
Villareal et al. [63]	107	70	BMI and PPT	1-year concurrent exercise with or without (control) hypocaloric diet	FFM (kg): −1.8, SE: 1.7 kg vs. −3.2, SE: 2.0 kg (control), *p* < 0.001 PPT (points): 5.4, SE: 2.4, *p* = 0.04
Villareal et al. [64]	160	70	BMI and PPT	Hypocaloric with RE, AE or RE + AE or isocaloric with no exercise (control)	FFM (kg): RE: −2.7, SE: 0.3, *p* < 0.01, AE: −2.7, SE: 0.3, *p* < 0.001, RE + AE: 1.7, SE: 0.3, *p* < 0.001 PPT (points): RE: 3.9, SE: 0.4, AE: 0.9, SE: 0.4, RE + AE: 5.5, SE: 0.4, all with *p* < 0.001
Kim et al. [91]	139	81	BF%, SMI and HGS	3-months CE with or without 3 gr supplementation of EAA or control	FM (kg): CE with EAA: −5.5 SE: 0.9, *p* = 0.036, CE without EAA: no sig. difference FFM (kg): no sig. differences

* Total number of subjects in studies is displayed. ** Intervention effects are displayed if a significant difference is reported compared to the control group. BMI—body mass index; SMM—skeletal muscle mass; BF%—body fat percentage; SMI—skeletal muscle index; VFA—visceral fat area; RE—resistance exercise, AE = aerobic exercise; CE—concurrent exercise; SPPB—short physical performance battery; HGS—handgrip strength; PT—peak torque; PPT—physical performance test; EAA—essential amino acids; SE—standard error; NP—not provided.
